# Genome-Wide Microsatellite Characterization and Marker Development in the Sequenced *Brassica* Crop Species

**DOI:** 10.1093/dnares/dst040

**Published:** 2013-10-14

**Authors:** Jiaqin Shi, Shunmou Huang, Jiepeng Zhan, Jingyin Yu, Xinfa Wang, Wei Hua, Shengyi Liu, Guihua Liu, Hanzhong Wang

**Affiliations:** Key Laboratory of Oil Crop Biology of the Ministry of Agriculture, Oil Crops Research Institute of the Chinese Academy of Agricultural Sciences, Wuhan 430062, China

**Keywords:** brassica, microsatellite, distribution, marker, database

## Abstract

Although much research has been conducted, the pattern of microsatellite distribution has remained ambiguous, and the development/utilization of microsatellite markers has still been limited/inefficient in *Brassica*, due to the lack of genome sequences. In view of this, we conducted genome-wide microsatellite characterization and marker development in three recently sequenced *Brassica* crops: *Brassica rapa*, *Brassica oleracea* and *Brassica napus*. The analysed microsatellite characteristics of these *Brassica* species were highly similar or almost identical, which suggests that the pattern of microsatellite distribution is likely conservative in *Brassica*. The genomic distribution of microsatellites was highly non-uniform and positively or negatively correlated with genes or transposable elements, respectively. Of the total of 115 869, 185 662 and 356 522 simple sequence repeat (SSR) markers developed with high frequencies (408.2, 343.8 and 356.2 per Mb or one every 2.45, 2.91 and 2.81 kb, respectively), most represented new SSR markers, the majority had determined physical positions, and a large number were genic or putative single-locus SSR markers. We also constructed a comprehensive database for the newly developed SSR markers, which was integrated with public *Brassica* SSR markers and annotated genome components. The genome-wide SSR markers developed in this study provide a useful tool to extend the annotated genome resources of sequenced *Brassica* species to genetic study/breeding in different *Brassica* species.

## 1. Introduction

Microsatellites, which are also known as simple sequence repeats (SSRs, often defined as 1–6 bp), variable numbers of tandem repeats (VNTRs) and short tandem repeats (STRs), have been found in all genomic regions of all examined organisms.^1^ Microsatellites have been traditionally regarded as ‘junk’ DNA and are mainly used as ‘neutral’ genetic markers.^2^ In recent years, microsatellites have been demonstrated to have many important biological functions (e.g. the regulation of chromatin organization, DNA metabolic processes, gene activity and RNA structure)^3,4^ and have therefore emerged as the third major class of genetic variations, alongside single nucleotide polymorphisms (SNPs) and copy number variations (CNVs).^5^ Microsatellite markers are co-dominant, multi-allelic, easily detected, hyper-variable, highly reproducible and abundant in the genome.^6^ Therefore, among the available genetic marker systems (e.g. RFLP, RAPD, SSR, AFLP, SRAP and SNP), the SSR marker has been the preferential choice for various applications, such as variety identification, genetic diversity evaluation, phylogenetic relationship analysis, genetic map construction, linkage/association mapping of gene/QTL, marker-assisted selection and comparative mapping.^7,8^

Of the 47 genera in the *Brassiceae* tribe within the *Brassicaceae* (*Cruciferae*) family, the genus *Brassica* currently comprises 38 species,^9^ which include economically important crops that provide many vegetables, condiments, fodders and oil products.^10^ The main cultivated *Brassica* species include three diploid species, *Brassica rapa* (AA, *n* = 10), *Brassica nigra* (BB, *n* = 8) and *Brassica oleracea* (CC, *n* = 9) and three allotetraploid species, *Brassica juncea* (AABB, *n* = 18), *Brassica napus* (AACC, *n* = 19) and *Brassica carinata* (BBCC, *n* = 17). The genetic relationship of the six widely cultivated *Brassica* species are described as U's triangle^11^ in which the three allotetraploid species originated from the chromosome doubling after the natural hybridization between the three diploid species.

Much research has been conducted to identify/characterize genomic/genic microsatellites and/or to develop markers in the *Brassica* species through probe (containing a repeated motif) hybridization against genomic/cDNA clones^12–19^ or through *in silico* analysis of publicly available bacterial artificial chromosome (BAC) sequences,^20^ BAC-end sequences (BESs),^21–23^ genome survey sequences (GSSs),^24^ whole genome shotgun sequences (WGSs),^25,26^ expressed sequence tag sequences^27,28^ and unique transcript sequences.^29–32^ However, the pattern of microsatellite distribution has remained ambiguous, and the development/utilization of SSR marker has still been limited/inefficient in *Brassica*, which is mostly due to the lack of genome sequences. First, the sequences, programmes, criteria and parameters that are used for mining microsatellites usually have differed across these previous studies, which have made it difficult to compare and integrate these results to obtain the definitive conclusions on the pattern of microsatellite distribution. Secondly, only a small part of the genomic sequences of usually one species have been analysed in each of these previous studies. Therefore, it has been impossible to obtain general conclusions on the pattern of microsatellite distribution. In addition, the total number (≈10 000) of previously developed publicly available SSR markers is still limited^33^ and not sufficient for many studies, which require a large number and/or high density of genetic markers, such as high-density linkage map construction, gene/QTL fine-mapping and genome-wide/regional association mapping. Thirdly, due to the lack of genome sequences, the genomic distribution of microsatellites and the physical position(s)/product(s) number of the previously developed publicly available *Brassica* SSR markers have been all or mostly unclear, which has hindered their exact and/or effective utilization.

Thanks to the rapid development of genome sequencing technology, the genome sequences are currently available for tens of plant species (http://www.phytozome.net), including three recently sequenced *Brassica* crop species, namely *B. rapa*,^34^ *B. oleracea* (http://www.ocri-genomics.org/bolbase/index.html) and *B. napus* (our unpublished data). These sequences provide a powerful tool for genome-wide microsatellite characterization and/or marker development, which has been conducted in several model and crop plants, such as *Arabidopsis* (http://www.arabidopsis.org/), rice,^35^ maize (mips.helmholtz-muenchen.de/plant/maize/), sorghum (genome.jgi-psf.org/Sorbi1/Sorbi1.home.htm), black cottonwood,^36^ cucumber,^37^ *Brachypodim distachyon*^38^ and foxtail millet^39^ but not *Brassica*. In view of this circumstance, we conducted genome-wide microsatellite characterization and marker development in the three sequenced *Brassica* crop species. The main objectives of this study were as follows: (i) to characterize and compare the frequency and distribution with respect to the motif length, type and repeat number of microsatellites in the assembled genomic sequences of these *Brassica* species; (ii) to characterize and compare the genomic distribution of microsatellites in the assembled pseudochromosomes of these *Brassica* species; (iii) to develop SSR markers from the assembled genomic sequences of these *Brassica* species and determine their copy number and positional relationship with the previously developed publicly available *Brassica* SSR markers and the annotated genome components; (iv) to construct a user-friendly comprehensive SSR marker database of *Brassica* and (v) to evaluate the newly developed genome-wide SSR markers by PCR (polymerase chain reaction) amplification in representative *B. napus* inbred lines.

## 2. Materials and methods

### 2.1. Sources of genome sequences

The three inbred/pure lines, namely Chiifu-401 (*B. rapa*), O212 (*B. oleracea*) and Zhongshuang11 (*B. napus*), were sequenced by our own and several other institutes using Illumina GA II technology, and high-quality sequence reads were assembled using stringent parameters. Finally, a total of 40 549 (283.8 Mb), 120 061 (540.0 Mb) and 5098 (1000.9 Mb) sequence scaffolds were obtained for *B. rapa*, *B. oleracea* and *B. napus*, respectively, which represents 58.5, 77.6 and 81.7% of the nuclear genome and covers >98% of the gene space.

### 2.2. Identification of microsatellites

PERL5 script MIcroSAtellite (http://pgrc.ipk-gatersleben.de/misa/)^40^ was used to identify and localize perfect microsatellites as well as compound microsatellites that are interrupted by a certain number of bases. The repeat unit length was defined as the default mono- to hexanucleotide because microsatellites of longer repeat units are very scarce. The minimum repeat unit was defined as 12, 6, 4, 3, 3 and 3, respectively, for the mono- to hexanucleotide. Compound microsatellites were defined as ≥2 repeats interrupted by ≤100 bp.

### 2.3. Development of SSR primers

Primer pairs were designed from the flanking sequences of identified microsatellites using the primer3_core program (http://www-genome.wi.mit.edu/cgi-bin/primer/primer3_ www.cgi) in batch mode. Two perl scripts, p3_in.pl and p3_out.pl, serve as interface modules for the programme-to-programme data interchange between MISA and the primer modeling software Primer3. The primer-designing parameters were 18–27 bp primer length, 57–63°C melting temperature, 30–70% GC content and 100–300 bp product size. The designed SSR primer pairs were denominated as the names of sequence scaffolds followed by a serial number of microsatellites (such as BrScaffold000001_1).

### 2.4. Localization/mapping of SSR markers by *in silico* PCR

The primer-pair sequences of previously developed publicly available *Brassica* SSR markers were downloaded from the brassica.info website (http://www.brassica.info/resource/markers/ssr-exchange.php) and additional files in the recent literature.^20,24,26,29,30,32^ To determine their physical positions and copy numbers, the previously and newly developed *Brassica* SSR markers were aligned to the assembled genomic sequences of the studied *Brassica* species. This alignment was conducted using the *in silico* PCR method^41^ with the following default parameters: 2 bp mismatch, 1 bp gap, 50 bp margin and 50–1000 bp product size.

### 2.5. Validation of SSR markers by PCR amplification

A total of 3974 SSR primer pairs were synthesized to test for PCR amplification in six representative *B. napus* cultivars/inbred lines (Tapidor, Westar, Zhongshuang11, No. 07197, No. 73290 and No. 91032), which were chosen from the core collections of a natural population and the parents of several segregating populations in our laboratory, for their large genetic distance and extreme trait(s) performance (our unpublished data).

Genomic DNA of the six accessions was isolated from young leaves. PCR was performed in 20-µl volume that contained 0.2 mM dNTP, 0.5 U of Taq DNA polymerase, 75 ng of template DNA, 0.5 µM each primer and 1× PCR buffer (10 mM Tris pH 9.0, 50 mM KCl and 1.5 mM MgCl_2_). DNA amplification was conducted by the ‘touchdown’ method, with the following thermal profile: initial denaturation at 94°C for 5 min; six cycles of 30 s at 94°C, 45 s at 63°C with a 1°C decrease in annealing temperature per cycle and 1 min at 72°C; 26 cycles of 30 s at 94°C, 45 s at 57°C and 1 min at 72°C and a final extension at 72°C for 10 min. The PCR products were separated on 6% denaturing polyacrylamide gels and were visualized by silver staining.

### 2.6. Statistical analysis

The correlation analysis was performed using the SAS PROC CORR procedure incorporated into SAS version 8.0. The Excel statistical function CHISQ.TEST was used to obtain the significance level ($P_{\chi ^2 \,{\rm test}} $) of the degree of fit for the practical and hypothetical distributions of microsatellites as well as genes and TEs in the assembled pseudochromosomes.

## 3. Results

### 3.1. Frequency and distribution with respect to the motif length, type and number

A total of 140 998, 229 389 and 420 991 perfect mono- to hexanucleotide repeat microsatellites were identified from 283.8, 540.0 and 1000.9 Mb of assembled genomic sequences of *B. rapa*, *B. oleracea* and *B. napus*, respectively (Table 1), with an overall frequency of 496.8, 424.8 and 420.6 per Mb or one every 2.01, 2.35 and 2.38 kb.

**Table 1.** Number, repeat number and total repeat length of the mono- to hexanucleotide repeats or motifs of microsatellites in the assembled genomic sequences of *B. rapa*, *B. oleracea* and *B. napus*

Motif	*B. rapa*			*B. oleracea*			*B. napus*		
	Number (%)	Repeat number	Total length (%)	Number (%)	Repeat number	Total length (%)	Number (%)	Repeat number	Total length (%)
Mono	31 258 (22.2)	12–307 (14.7)	458 968 (20.1)	55 433 (24.2)	3–65 (15.1)	838 104 (24.1)	97 128 (23.1)	12–2545 (15.2)	147 5939 (22.9)
A	29 536 (20.9)	12–50 (14.5)	428 733 (18.7)	52 021 (22.7)	3–65 (15.0)	780 171 (22.5)	94 281 (22.4)	12–2545 (15.2)	1 432 867 (22.2)
C	1722 (1.2)	12–307 (17.6)	30 235 (1.3)	3412 (1.5)	3–63 (17.0)	57 933 (1.7)	2847 (0.7)	12–83 (15.1)	43 072 (0.7)
Di	33 885 (24.0)	6–3644 (11.1)	751 910 (32.9)	55 336 (24.1)	6–82 (8.8)	968 946 (27.9)	98 816 (23.5)	6–5556 (9.1)	1 789 752 (27.8)
AT	19 697 (14.0)	6–419 (8.9)	350 590 (15.3)	33 315 (14.5)	6–63 (8.9)	596 070 (17.2)	57 070 (13.6)	6–5556 (8.7)	996 518 (15.5)
AG	11 683 (8.3)	6–3644 (15.6)	364 004 (15.9)	18 593 (8.1)	6–44 (8.7)	322 438 (9.3)	34 638 (8.2)	6–1732 (9.9)	688 124 (10.7)
AC	2490 (1.8)	6–131 (7.5)	37 124 (1.6)	3411 (1.5)	6–82 (7.4)	50 220 (1.4)	7072 (1.7)	6–92 (7.4)	104 648 (1.6)
CG	15 (0.0)	6–8 (6.4)	192 (0.0)	17 (0.0)	6–9 (6.4)	218 (0.0)	36 (0.0)	6–8 (6.4)	462 (0.0)
Tri	32 387 (23.0)	4–812 (4.7)	459 039 (20.1)	47 716 (20.8)	4–1710 (4.7)	670 164 (19.3)	91 448 (21.7)	4–1794 (4.7)	1 277 172 (19.8)
AAG	9796 (6.9)	4–812 (4.7)	139 239 (6.1)	15 322 (6.7)	4–81 (4.6)	212 973 (6.1)	29 395 (7.0)	4–162 (4.6)	408 264 (6.3)
AAT	6334 (4.5)	4–573 (4.9)	93 240 (4.1)	9355 (4.1)	4–1710 (5.2)	145 056 (4.2)	17 722 (4.2)	4–1794 (5.1)	268 956 (4.2)
ATC	4211 (3.0)	4–190 (5.0)	63 213 (2.8)	6093 (2.7)	4–81 (4.7)	85 047 (2.4)	12 314 (2.9)	4–119 (4.6)	170 151 (2.6)
AAC	3637 (2.6)	4–11 (4.5)	49 146 (2.1)	5036 (2.2)	4–41 (4.4)	66 705 (1.9)	10 046 (2.4)	4–22 (4.5)	134 592 (2.1)
AGG	3243 (2.3)	4–179 (4.6)	45 084 (2.0)	5425 (2.4)	4–13 (4.6)	74 763 (2.2)	8349 (2.0)	4–14 (4.6)	115 494 (1.8)
ACC	2144 (1.5)	4–9 (4.5)	28 908 (1.3)	2788 (1.2)	4–43 (4.4)	37 155 (1.1)	5969 (1.4)	4–14 (4.4)	79 137 (1.2)
AGC	1127 (0.8)	4–11 (4.4)	15 039 (0.7)	1390 (0.6)	4–22 (4.4)	18 507 (0.5)	2878 (0.7)	4–18 (4.4)	38 379 (0.6)
ACT	674 (0.5)	4–10 (4.4)	8874 (0.4)	818 (0.4)	4–28 (4.5)	10 968 (0.3)	1754 (0.4)	4–17 (4.4)	23 265 (0.4)
CCG	626 (0.4)	4–8 (4.4)	8220 (0.4)	799 (0.3)	4–7 (4.3)	10 203 (0.3)	1566 (0.4)	4–8 (4.3)	20 139 (0.3)
ACG	595 (0.4)	4–9 (4.5)	8076 (0.4)	690 (0.3)	4–13 (4.2)	8787 (0.3)	1455 (0.3)	4–10 (4.3)	18 795 (0.3)
Tetra	29 433 (20.9)	3–264 (3.2)	376 668 (16.5)	48 394 (21.1)	3–54 (3.1)	608 452 (17.5)	91 268 (21.7)	3–631 (3.1)	1 148 496 (17.8)
AAAT	11 870 (8.4)	3–8 (3.2)	152 776 (6.7)	18 856 (8.2)	3–28 (3.1)	236 560 (6.8)	33 903 (8.1)	3–10 (3.1)	425 080 (6.6)
AAAG	3496 (2.5)	3–60 (3.2)	44 608 (1.9)	5697 (2.5)	3–36 (3.2)	72 224 (2.1)	10 795 (2.6)	3–13 (3.2)	136 204 (2.1)
AAAC	3333 (2.4)	3–8 (3.1)	41 960 (1.8)	4723 (2.1)	3–26 (3.1)	59 076 (1.7)	9717 (2.3)	3–8 (3.1)	121 768 (1.9)
AATT	2534 (1.8)	3–6 (3.1)	31 108 (1.4)	4088 (1.8)	3–10 (3.1)	51 416 (1.5)	7863 (1.9)	3–41 (3.1)	98 224 (1.5)
AATC	1137 (0.8)	3–8 (3.2)	14 368 (0.6)	2956 (1.3)	3–27 (3.1)	36 908 (1.1)	5112 (1.2)	3–8 (3.1)	63 884 (1.0)
others	7063 (5.0)	3–264 (3.3)	91 848 (4.0)	12 074 (5.3)	3–54 (3.2)	152 268 (4.4)	23 878 (5.7)	3–631 (3.2)	303 336 (4.7)
Penta	9856 (7.0)	3–114 (3.2)	156 510 (6.8)	15 012 (6.5)	3–40 (3.2)	241 090 (6.9)	29 058 (6.9)	3–28 (3.1)	457 065 (7.1)
AAAAT	2758 (2.0)	3–63 (3.1)	42 920 (1.9)	4051 (1.8)	3–18 (3.4)	67 905 (2.0)	7617 (1.8)	3–9 (3.1)	118 030 (1.8)
AACCG	1000 (0.7)	3–68 (3.3)	16 510 (0.7)	2169 (0.9)	3–40 (3.2)	34 645 (1.0)	3541 (0.8)	3–7 (3.2)	56 435 (0.9)
AAAAC	878 (0.6)	3–7 (3.2)	13 905 (0.6)	1186 (0.5)	3–8 (3.2)	18 715 (0.5)	2424 (0.6)	3–10 (3.2)	38 280 (0.6)
AAAAG	683 (0.5)	3–7 (3.1)	10 675 (0.5)	1164 (0.5)	3–14 (3.1)	18 260 (0.5)	2351 (0.6)	3–7 (3.1)	36 820 (0.6)
AAATT	568 (0.4)	3–6 (3.1)	8790 (0.4)	784 (0.3)	3–5 (3.1)	12 030 (0.3)	1488 (0.4)	3–7 (3.1)	23 065 (0.4)
AAACC	490 (0.3)	3–7 (3.2)	7850 (0.3)	713 (0.3)	3–6 (3.2)	11 325 (0.3)	1273 (0.3)	3–9 (3.2)	20 290 (0.3)
AATAT	394 (0.3)	3–6 (3.1)	6085 (0.3)	573 (0.2)	3–6 (3.1)	8890 (0.3)	1083 (0.3)	3–13 (3.1)	16 905 (0.3)
Others	3085 (2.2)	3–114 (3.2)	49 775 (2.2)	4372 (1.9)	3–16 (3.2)	69 320 (2.0)	9281 (2.2)	3–28 (3.2)	147 240 (2.3)
Hexa	4179 (3.0)	3–102 (3.4)	85 176 (3.7)	7498 (3.3)	3–33 (3.3)	146 370 (4.2)	13 273 (3.2)	3–1579 (3.7)	295 236 (4.6)
AAAAAT	628 (0.4)	3–52 (3.2)	12 000 (0.5)	987 (0.4)	3–22 (3.1)	18 618 (0.5)	1803 (0.4)	3–35 (3.1)	33 972 (0.5)
AAAATT	267 (0.2)	3–6 (3.0)	4866 (0.2)	776 (0.3)	3–25 (3.1)	14 376 (0.4)	1341 (0.3)	3–154 (3.2)	25 614 (0.4)
AAAAAC	269 (0.2)	3–23 (3.3)	5298 (0.2)	395 (0.2)	3–7 (3.2)	7596 (0.2)	746 (0.2)	3–22 (3.3)	14 562 (0.2)
AAAAAG	175 (0.1)	3–6 (3.2)	3330 (0.1)	357 (0.2)	3–14 (3.3)	6966 (0.2)	644 (0.2)	3–37 (3.3)	12 612 (0.2)
AAATAT	150 (0.1)	3–52 (3.8)	3390 (0.1)	246 (0.1)	3–16 (3.2)	4704 (0.1)	436 (0.1)	3–29 (3.3)	8562 (0.1)
Others	2690 (1.9)	3–102 (3.5)	56 292 (2.5)	4737 (2.1)	3–33 (3.3)	94 110 (2.7)	8303 (2.0)	3–1579 (4.0)	199 914 (3.1)
Total	140 998 (100)	3–3644 (8.0)	228 8271 (100)	229 389 (100)	3–1710 (7.7)	347 3126 (100)	420 991 (100)	3–5556 (7.7)	6 443 660 (100)

**Figure 1.**
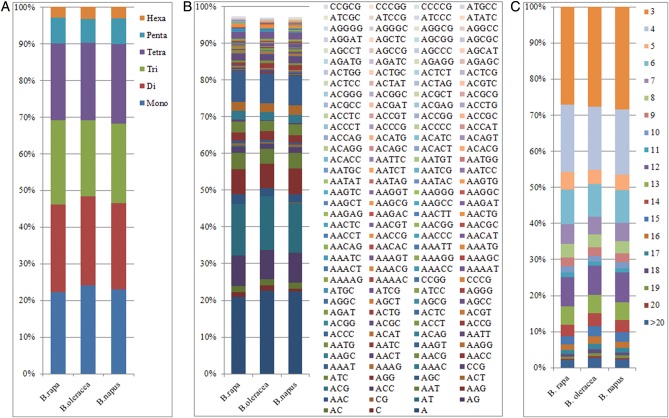
Distribution with respect to the motif length (A), type (B) and repeat number (C) of microsatellites in the assembled genomic sequences of *B. rapa*, *B. oleracea* and *B. napus*. The vertical axis shows the abundances (%) of microsatellites with different motif lengths, types or repeat numbers that are discriminated by the legends of different colours. For Figure 1B, because there is a limitation in the number of items (<256) in Excel, the abundances of the mono- to pentanucleotide motifs (a total of 151 types) are shown, while those for the hexanucleotide motifs (a total of 350 types) are displayed in Supplementary Table S2.

In accordance with their high correlation (Supplementary Table S1), the distributions with respect to the motif length of microsatellites in the assembled genomic sequences of *B. rapa*, *B. oleracea* and *B. napus* were almost identical: mono-, di-, tri- and tetranucleotide repeats accounted for very similar and relatively high proportions, whereas penta- and hexanucleotide repeats were relatively uncommon (Fig. 1A).

In accordance with their high correlation (Supplementary Table S1), the distributions with respect to the motif type of microsatellites in the assembled genomic sequences of *B. rapa*, *B. oleracea* and *B. napus* were almost identical (Fig. 1B; Supplementary Table S2). More specifically, both the dominant/major and absent/scarce mono- to hexanucleotide motifs in the assembled genomic sequences of the three *Brassica* species were mostly identical (Table 1; Supplementary Table S3). Interestingly, the dominant/major motifs (A, AT, AAG/AAT, AAAT, AAAAT and AAAAAT) were all A/T rich (Table 1), whereas the absent/scarce motifs were mostly C/G rich (Supplementary Table S3), which were highly consistent with the previous reports on microsatellites identified from 536 seed BACs of *B. rapa*,^20^ 3500 genomic clones^42^ and 595 577 WGSs^26^ of *B. oleracea* and 13 794 GSSs (mainly BESs) of *B. napus.*^24^ It should be noted that the nucleotide composition characteristics of both the dominant/major and absent/scarce motifs in the assembled genomic sequences of the three *Brassica* species corresponded well to their much higher A/T (mean = 63.8%) than C/G (mean = 36.2%) content.

**Figure 2.**
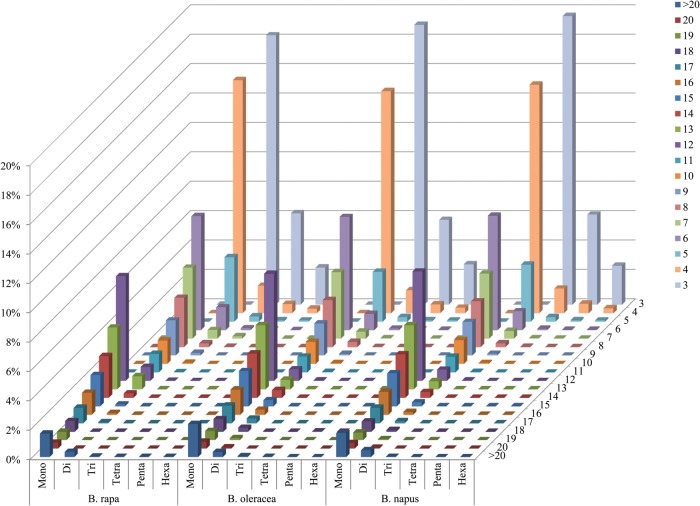
Distribution with respect to the motif repeat number of the individual mono- to hexanucleotide repeat microsatellites in the assembled genomic sequences of *B. rapa*, *B. oleracea* and *B. napus*. The vertical axis shows the abundances of microsatellites that have different motif repeat numbers (from 3 to >20), which are discriminated by legends of different colours.

In accordance with their high correlation (Supplementary Table S1), the distributions with respect to the motif repeat number of microsatellites in the assembled genomic sequences of *B. rapa*, *B. oleracea* and *B. napus* were also almost identical (Fig. 1C). Obviously, the microsatellite abundances decreased significantly as the motif repeat number increased, and the rate of this change was the slowest for dinucleotide repeat, followed by mono- and trinucleotide repeats, and was faster for other long repeats (Fig. 2). As a consequence, the difference between the average and minimum motif repeat numbers was the largest for dinucleotide repeat, followed by mono- and trinucleotide repeats, and was relatively small for tetra- to hexanucleotide repeats (Table 1).

In addition, the motif repeat number of the corresponding mono- to hexanucleotide repeats or motifs of microsatellites in the assembled genomic sequences of *B. rapa*, *B. oleracea* and *B. napus* were highly similar (Table 1; Supplementary Table S4). As a consequence, the total repeat length (=microsatellite number × motif length × motif repeat number) proportions of the corresponding mono- to hexanucleotide repeats or motifs of microsatellites in the assembled genomic sequences of *B. rapa*, *B. oleracea* and *B. napus* were mostly similar (Table 1; Supplementary Table S5).

### 3.2. Genomic distribution

**Figure 3.**
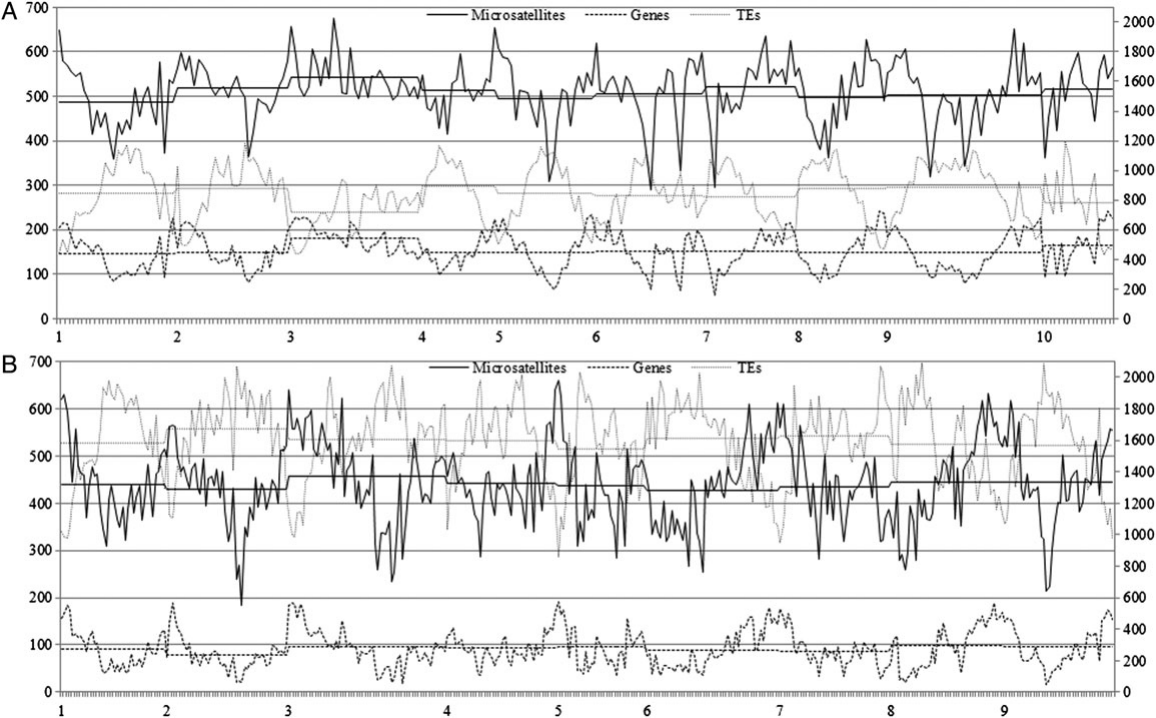
Genome-wide distributions of microsatellites as well as genes and TEs in the assembled pseudochromosomes of *B. rapa* (A) and *B. oleracea* (B). The horizontal axis shows the pseudochromosomes (*B. rapa*: A1–A10; *B. oleracea*: C1–C9), which are divided into 1-Mb intervals. The left and right vertical axes show the frequencies of the microsatellites/genes and TEs, respectively. On the figure, the curves/lines of different styles represent the practical/hypothetical(average) frequencies of microsatellites, genes and TEs, respectively.

The genomic distributions of microsatellites and their relation with the annotated genome components (mainly as genes and TEs) were investigated (Fig. 3; Table 2), based on the assembled pseudochromosomes of the sequenced *Brassica* species (currently available for *B. rapa* and *B. oleracea*; Supplementary Table S6).

**Table 2.** χ/italic>^2^ test between the practical and hypothetical/average distribution of microsatellites and their correlation with genes and TEs, for all pseudochromosomes of *B. rapa* and *B. oleracea*

Species	Chromosome	Microsatellites				Genes		TEs	
		Frequency	$P_{\chi ^2 \,{\rm test}} $	*r*_gene_	*r*_TE_	Frequency	$P_{\chi ^2 \,{\rm test}} $	Frequency	$P_{\chi ^2 \,{\rm test}} $
	A01	488	1.9E−42	0.76	−0.69	146	8.7E−60	841	0.0E+00
	A02	522	1.6E−16	0.78	−0.65	150	8.3E−41	869	4.0E−220
	A03	539	1.2E−13	0.46	−0.29	179	8.9E−15	725	9.9E−163
	A04	515	6.5E−14	0.53	−0.49	149	1.2E−17	887	1.9E−134
	A05	500	9.8E−37	0.77	−0.69	153	1.8E−63	828	2.9E−249
*B. rapa*	A06	509	6.1E−49	0.81	−0.58	153	7.6E−45	828	7.6E−122
	A07	524	5.2E−32	0.79	−0.65	155	2.3E−38	810	1.6E−173
	A08	502	1.8E−34	0.74	−0.66	150	1.8E−56	867	5.0E−206
	A09	505	4.3E−58	0.78	−0.63	150	9.2E−77	884	3.1E−255
	A10	522	6.1E−20	0.75	−0.57	166	6.5E−36	767	3.0E−210
	Total	512	2.4E−287	0.75	−0.61	155	0.0E+00	831	0.0E+00
	C01	442	1.5E−81	0.90	−0.82	93	1.7E−112	1570	0.0E+00
	C02	429	8.2E−108	0.81	−0.74	78	2.4E−103	1673	6.2E−248
	C03	459	1.4E−190	0.85	−0.71	96	1.0E−174	1606	0.0E+00
	C04	446	6.2E−59	0.86	−0.62	95	1.8E−48	1590	2.2E−258
*B. oleracea*	C05	439	1.6E−93	0.94	−0.84	97	4.2E−100	1535	8.2E−288
	C06	430	2.3E−139	0.90	−0.79	89	4.0E−150	1606	0.0E+00
	C07	435	4.7E−83	0.88	−0.74	86	6.6E−92	1630	3.5E−240
	C08	448	4.0E−163	0.87	−0.72	99	2.1E−173	1566	0.0E+00
	C09	445	3.8E−124	0.86	−0.73	96	3.3E−127	1566	0.0E+00
	Total	442	0.0E+00	0.87	−0.73	92	0.0E+00	1596	0.0E+00

For both *B. rapa* and *B. oleracea*, the frequency of microsatellites was high at/near both ends but low in/near the middle of all the pseudochromosomes (Fig. 3), which likely corresponded to the peri- telomere and centromere, respectively.^43^ The frequencies of microsatellites for the different pseudochromosomes of *B. rapa* or *B. oleracea* were generally comparable, which was in accordance with the similar frequencies of genes/TEs for these chromosomes (Fig. 3; Table 2). Interestingly, the homoeologous chromosomes A3 and C3 both exhibited the highest microsatellite frequency among all the pseudochromosomes of *B. rapa* or *B. oleracea*, respectively, which was in accordance with their highest gene frequency among these chromosomes (Fig. 3; Table 2). In accordance with the high significance of the *P*-values of the *χ*^2^ test between the practical and hypothetical/average frequencies of microsatellites in the 1-Mb genomic intervals (Table 2), the physical distribution of microsatellites on all the pseudochromosomes of both *B. rapa* and *B. oleracea* were highly non-uniform (Fig. 3), which suggests the non-random occurrence of microsatellites. In accordance with the usually higher *P*-values of the *χ*^2^ test between the practical and hypothetical/average frequencies of microsatellites for the 9 pseudochromosomes of *B. oleracea* than for the 10 pseudochromosomes of *B. rapa* (Table 2), the distribution of microsatellites was more uneven in *B. oleracea* than in *B. rapa* (Fig. 3), which was likely attributable to the more concentrated distribution of genes/TEs in *B. rapa* than in *B. oleracea*. For both *B. rapa* and *B. oleracea*, the frequencies of microsatellites in the 1-Mb genomic intervals studied were significantly positively or negatively correlated with those of genes (total *r* = 0.75 and 0.87) or TEs (total *r* = −0.61 and −0.73), respectively (Table 2), which was accordant with one of the interesting findings in this study, that the genomic distribution of microsatellites was generally in accordance with that of genes but opposite to that of TEs (Fig. 3). These results were in agreement with the previous findings, which showed that microsatellites are preferentially associated with non-repetitive DNA/gene sequences in the plant genome.^5,44^ The high agreement of microsatellites and genes strongly suggests the putative role of microsatellites in regulating gene function^3–5^ and the use of SSR markers for tagging/cloning genes.

In conclusion, the genomic distributions of microsatellites in the assembled pseudochromosomes of *B. rapa* and *B. oleracea* were generally similar.

### 3.3. Development and database of genome-wide SSR markers

A total of 115 869 (92.1%), 185 662 (91.4%) and 356 522 (95.0%) primer pairs were successfully designed from the flanking sequences of 125 856, 203 161 and 375 214 mono- to hexanucleotide and compound microsatellites identified from the assembled genomic sequences of *B. rapa*, *B. oleracea* and *B. napus*, respectively (Table 3). The primer pairs could not be designed for the remaining microsatellites, mostly due to the constraint of obtaining sufficient flanking sequences from either side of the identified microsatellites. Similar observations have also been observed in other genome-wide microsatellite marker development studies in plants, such as rice,^35^ black cottonwood,^36^ cucumber,^37^ *Brachypodim distachyon*^38^ and foxtail millet.^39^ The frequencies of newly developed genome-wide SSR markers of *B. rapa*, *B. oleracea* and *B. napus* were 408.2, 343.8 and 356.2 per Mb or one every 2.45, 2.91 and 2.81 kb, respectively. Most of the genome-wide SSR markers of *B. rapa* (91.9%) and *B. oleracea* (75.4%) were developed from the mapped sequence scaffolds and thus have determined physical positions. The physical positions of the newly developed genome-wide SSR markers of *B. napus* will be determined soon because the anchoring of its sequence scaffolds will be completed after several months (our unpublished data).

**Table 3.** Number (%) of newly developed genome-wide SSR markers that generated certain numbers (from 0 to >3) of *in silico* PCR products in the assembled genomic sequences of *B. rapa*, *B. oleracea* and *B. napus*, respectively

Markers from	*In silico* PCR in	Zero	One	Two	Three	>Three	Total
	*B. rapa*	47 (0.0)	92 517 (79.8)	10 977 (9.5)	3309 (2.9)	9019 (7.8)	
*B. rapa*	*B. oleracea*	59 201 (51.1)	40 493 (34.9)	6370 (5.5)	2100 (1.8)	7705 (6.6)	115 869 (100)
	*B. napus*	12 765 (11.0)	36 030 (31.1)	38 162 (32.9)	10 666 (9.2)	18 246 (15.7)	
	*B. rapa*	110 784 (59.7)	48 423 (26.1)	8486 (4.6)	3349 (1.8)	14 620 (7.9)	
*B. oleracea*	*B. oleracea*	20 (0.0)	121 169 (65.3)	18 299 (9.9)	7140 (3.8)	39 034 (21.0)	185 662 (100)
	*B. napus*	13 192 (7.1)	54 749 (29.5)	55 160 (29.7)	14 156 (7.6)	48 405 (26.1)	
	*B. rapa*	157 473 (44.2)	138 202 (38.8)	20 604 (5.8)	7758 (2.2)	32 485 (9.1)	
*B. napus*	*B. oleracea*	106 706 (29.9)	153 690 (43.1)	24 601 (6.9)	10 264 (2.9)	61 261 (17.2)	356 522 (100)
	*B. napus*	0 (0.0)	93 084 (26.1)	110 106 (30.9)	44 138 (12.4)	109 194 (30.6)	

Because of the polyploidy nature of *Brassica*,^45^ SSR markers usually amplify multiple fragments from homologous DNA sequences, which could complicate or cause errors in the genotype scoring. Therefore, all of the newly developed genome-wide SSR markers were subjected to *in silico* PCR analysis in the assembled genomic sequences of *B. rapa*, *B. oleracea* and *B. napus*, and the numbers of *in silico* PCR product(s) were recorded and summarized (Table 3). For the 115 869 SSR markers developed from *B. rapa*, 47 (0.0%), 92 517 (79.8%), 10 977 (9.5%), 3309 (2.9%) and 9019 (7.8%) markers generated 0, 1, 2, 3 and >3 *in silico* PCR product(s), respectively, from the assembled genomic sequences of *B. rapa*; 59 201 (51.1%), 40 493 (34.9%), 6370 (5.5%), 2100 (1.8%) and 7705 (6.6%) markers generated 0, 1, 2, 3 and >3 *in silico* PCR product(s), respectively, from the assembled genomic sequences of *B. oleracea* and 12 765 (11.0%), 36 030 (31.1%), 38 162 (32.9%), 10 666 (9.2%) and 18 246 (15.7%) markers generated 0, 1, 2, 3 and >3 *in silico* PCR product(s), respectively, from the assembled genomic sequences of *B. napus.* For the 185 662 SSR markers developed from *B. oleracea*, 20 (0.0%), 121 169 (65.3%), 18 299 (9.9%), 7140 (3.8%) and 39 034 (21.0%) markers generated 0, 1, 2, 3 and >3 *in silico* PCR product(s), respectively, from the assembled genomic sequences of *B. oleracea*; 110 784 (59.7%), 48 423 (26.1%), 8486 (4.6%), 3349 (1.8%) and 14 620 (7.9%) markers generated 0, 1, 2, 3 and >3 *in silico* PCR product(s), respectively, from the assembled genomic sequences of *B. rapa* and 13 192 (7.1%), 54 749 (29.5%), 55 160 (29.7%), 14 156 (7.6%) and 48 405 (26.1%) markers generated 0, 1, 2, 3 and >3 *in silico* PCR product(s), respectively, from the assembled genomic sequences of *B. napus.* For the 356 522 SSR markers developed from *B. napus*, 0 (0.0%), 93 084 (26.1%), 110 106 (30.9%), 44 138 (12.4%) and 109 194 (30.6%) markers generated 0, 1, 2, 3 and >3 *in silico* PCR product(s), respectively, from the assembled genomic sequences of *B. napus*; 157 473 (44.2%), 138 202 (38.8%), 20 604 (5.8%), 7758 (2.2%) and 32 485 (9.1%) markers generated 0, 1, 2, 3 and >3 *in silico* PCR product(s), respectively, from the assembled genomic sequences of *B. rapa* and 106 706 (29.9%), 153 690 (43.1%), 24 601 (6.9%), 10 264 (2.9%) and 61 261 (17.2%) markers generated 0, 1, 2, 3 and >3 *in silico* PCR product(s), respectively, from the assembled genomic sequences of *B. oleracea*. Interestingly, the SSR markers that generated tens to thousands of *in silico* PCR products were mostly associated with the annotated TEs, especially the retrotransposons.

We also determined the relationship between the physical positions of the newly developed genome-wide SSR markers and the previously developed publicly available *Brassica* SSR markers as well as the annotated genome components (mainly as genes and TEs) (Supplementary Table S7). Of the 115 869 SSR markers developed from *B. rapa*, 5991 (5.2%), 22 596 (19.5%) and 32 648 (28.2%) were involved in public *Brassica* SSR markers, genes and TEs, respectively. Of the 185 662 SSR markers developed from *B. oleracea*, 12 322 (6.6%), 33 228 (17.9%) and 73 487 (39.6%) were involved in public *Brassica* SSR markers, genes and TEs, respectively. Of the 356 522 SSR markers developed from *B. napus*, 23 928 (6.7%), 58 952 (16.5%) and 161 090 (45.2%) were involved in public *Brassica* SSR markers, genes and TEs, respectively. Interestingly, the TE-associated SSR markers were rarely involved in the annotated genes and mostly generated tens to thousands of *in silico* PCR products.

**Figure 4.**
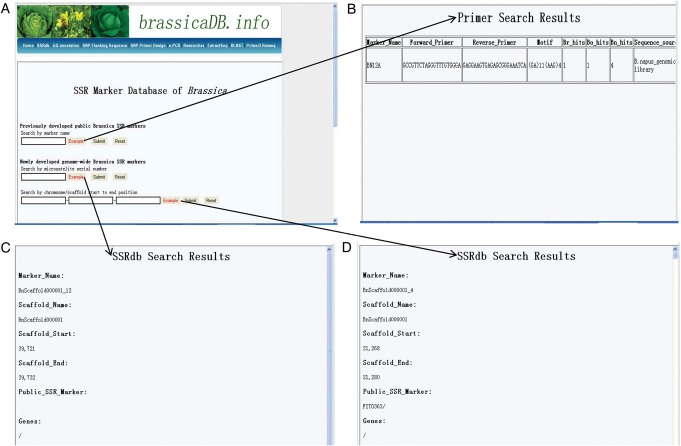
*Brassica* SSR marker database homepage (A) and search results pages (B–D). This database provides three search approaches: by the name of the previously developed publicly available *Brassica* SSR markers (e.g. BN12A), by the serial number of the SSR markers (e.g. BnScaffold000001_12) and by the start to end positions on the chromosome/scaffold (e.g. BnScaffold000001:20–20 000 bp). This database also provides many useful tools, such as e-PCR, BLAST and primer3. Figure B–D show the results of examples of the three search approaches that are provided in the homepage.

To facilitate the access and effective utilization of the *Brassica* SSR markers, we constructed an integrative database (http://oilcrops.info/SSRdb), which has search tools to obtain much useful information for the newly developed genome-wide SSR markers from the sequenced *Brassica* species and the previously developed publicly available *Brassica* SSR markers (Fig. 4). For the previously developed publicly available *Brassica* SSR markers, this information includes the primer-pair sequences, microsatellite repeat, source, reference and number of *in silico* PCR product(s) in the assembled genomic sequences of the sequenced *Brassica* species (currently only for *B. rapa*, *B. oleracea* and *B. napus*). For the newly developed genome-wide SSR markers from the sequenced *Brassica* species, this information includes the following: (i) the sequence, type, length and physical position of microsatellite repeat; (ii) the serial number, sequences, annealing temperatures, lengths and expected product size of primer pair; (iii) the number of *in silico* PCR product(s) in the assembled genomic sequences of the sequenced *Brassica* species (currently only for *B. rapa*, *B. oleracea* and *B. napus*) and (iv) the positional relationship with the previously developed publicly available *Brassica* SSR markers and the annotated genome components (mainly genes and TEs). In addition, this database also provides useful analysis tools (such as BLAST, e-PCR, Primer3 and ExtractSeq) and web links to other databases (e.g. http://brassicadb.org/brad/) and websites (e.g. http://www.brassica.info/) related to *Brassica* research. More importantly, this SSR marker database for *Brassica* will update as the number of sequenced *Brassica* species increases.

### 3.4. Experimental evaluation of the newly developed genome-wide SSR markers

A total of 3974 newly developed SSR markers from *B. rapa* and *B. oleracea* were tested for amplification in the six representative *B. napus* cultivars/inbred lines (Table 4). Of these, 3880 SSR markers (97.6%) successfully amplified at least one clear fragment, while the remaining 94 (2.4%) failed to amplify, which could be due to the differences between the genome sequences of *B. napus* and its two progenitors, *B. rapa* and *B. oleracea*.^46,47^ The amplification rate (97.6%) of the tested SSR markers in the six *B. napus* cultivars/inbred lines was slightly or much higher than the corresponding rates (94.3/82.9, 89.2 and 77.4%, respectively) for the previously developed SSR markers from GSSs (mainly BESs)/unique transcripts of *B. napus*,^24,30^ BACs of *B. rapa*^20^ and WGSs of *B. oleracea*,^26^ which suggests that there is a high quality in the SSR markers that were developed from the assembled genomic sequences. The amplification rate of the tested SSR markers showed small variations for different motif lengths, motif repeat numbers and repeat lengths (i.e. motif length × motif repeat number), which was consistent with the previous reports in *Brassica*^20,24,26^ and rice.^35^ For the majority of the tested SSR markers, the numbers of fragment(s) amplified from the six representative *B. napus* cultivars/inbred lines were equal or very close to those of *in silico* PCR product(s) in the assembled genomic sequences of *B. napus* (Supplementary Table S8). In particular, most (1602 of 1813; 88.4%) of the tested SSR markers that generated one *in silico* PCR product in the assembled genomic sequences of *B. napus* also amplified only a single clear fragment from the six representative *B. napus* cultivars/inbred lines. A considerable proportion (1099 of 3880; 28.3%) of the successfully amplified SSR markers also produced weak fragment(s), which could correspond to non-specific amplification(s) from homologous DNA sequences.

**Table 4.** Amplification and polymorphism rate of the tested SSR markers and their association with the number of amplified fragment(s), the motif length, the motif repeat number and the repeat length

		Total markers		Amplified markers		Polymorphic markers	
		Number	%	Number	%	Number	%
	0	94	2.4	/	/	/	/
	1	1859	46.8	/	/	1255	67.5
Fragment (s) number	2	1541	38.8	/	/	1124	72.9
	3	352	8.9	/	/	276	78.4
	>3	128	3.2	/	/	110	85.9
	Mono	35	0.9	34	97.1	26	76.5
	Di	3156	79.4	3077	97.5	2195	71.3
	Tri	594	14.9	584	98.3	397	68.0
Motif length	Tetra	74	1.9	73	98.6	41	56.2
	Penta	21	0.5	20	95.2	15	75.0
	Hexa	12	0.3	12	100.0	11	91.7
	Compound	82	2.1	80	97.6	80	100.0
	5	496	12.5	488	98.4	316	64.8
	6	98	2.5	96	98.0	67	69.8
	7	59	1.5	57	96.6	41	71.9
	10	791	19.9	765	96.7	539	70.5
	11	528	13.3	513	97.2	347	67.6
Motif repeat number	12	388	9.8	375	96.6	252	67.2
	13	576	14.5	567	98.4	386	68.1
	14	295	7.4	288	97.6	209	72.6
	15	98	2.5	95	96.9	76	80.0
	16	140	3.5	139	99.3	114	82.0
	17	105	2.6	105	100.0	88	83.8
	Others	400	10.1	392	98.0	330	84.2
	15	412	10.4	405	98.3	266	65.7
	18	82	2.1	80	97.6	55	68.8
	20	840	21.1	814	96.9	561	68.9
	21	58	1.5	56	96.6	40	71.4
	22	526	13.2	511	97.1	344	67.3
Repeat length	24	418	10.5	405	96.9	275	67.9
	26	577	14.5	568	98.4	387	68.1
	28	303	7.6	295	97.4	213	72.2
	30	122	3.1	119	97.5	96	80.7
	32	133	3.3	132	99.2	107	81.1
	34	97	2.4	97	100.0	80	82.5
	Others	406	10.2	398	98.0	341	85.7
Total		3974	100	3880	97.6	2765	71.3

The majority (2765 of 3880; 71.3%) of the successfully amplified SSR markers was polymorphic across the six representative *B. napus* cultivars/inbred lines (Table 4). The polymorphism rate of the tested SSR markers was almost equal to or slightly higher than the corresponding rates (73.4/65.9, 57.9, 69.5%) for the previously developed SSR markers from GSSs (mainly BESs)/unique transcripts of *B. napus*,^24,30^ BACs of *B. rapa*^20^ and WGSs of *B. oleracea*.^26^ Obviously, the polymorphism rate of the tested SSR markers increased (from 67.5% to 85.9%) as the number of amplified bands increased (from 1 to >3). The polymorphism rate of the tested SSR markers decreased slightly from the mono- to tetranucleotide repeats, while it increased quickly from the penta- to hexanucleotide repeats. This inconsistency of the relationship between the SSR marker polymorphism level and the motif length was also observed frequently in the previous SSR marker evaluation experiments, such as in the tests of the 627 and 1000 SSR markers from the GSSs (mainly BESs) and unique transcripts, respectively, of *B. napus*,^24,30^ the 890 SSR markers from the BACs of *B. rapa*,^20^ the 1398 SSR markers from the WGSs of *B. oleracea*^26^ and the 1009 SSR markers from the assembled genomic sequences of cucumber.^37^ This type of inconsistency could be attributable to the observation that only a small number of SSR markers of the specific (usually long) motif length(s) have been used to investigate this relationship in all of the above-mentioned studies (e.g. only 21 and 12 penta- and hexanucleotide repeat SSR markers were tested in the current investigation), which worthwhile to develop more SSR markers with long motifs to further investigate the relationship between the SSR marker polymorphism level and the motif length. The polymorphism rate of the tested SSR markers was highly positively correlated with both the motif repeat number and the repeat length (*r* = 0.74 and 0.86, respectively), which was basically consistent with the previous reports in *Brassica*^24,30^ and other plant species, including cucumber^37^ and carrot.^48^ Both correlation coefficients in the current investigation were much higher than or equal to the corresponding values (0.21 and 0.41; 0.74 and _) that were estimated with the 627 SSR markers from the GSSs (mainly BESs) of *B. napus*^24^ or the 1009 SSR markers from the assembled genomic sequences of cucumber,^37^ respectively. Strikingly, the tested SSR markers that were designed from compound repeats were almost all (80 of 82; 97.6%) polymorphic across the six representative *B. napus* cultivars/inbred lines (Supplementary Table S8).

Because the 1055 and 2919 tested SSR markers were developed from the sequence scaffolds of *B. rapa* and *B. oleracea*, respectively, they were thus designated as ‘BrSF’ and ‘BoSF’. To facilitate the effective utilization of these tested newly developed BrSF and BoSF SSR markers, the following useful information was provided (Supplementary Table S8): (i) the type, length, position and sequence of the microsatellite repeat; (ii) the name, sequences, annealing temperatures and expected product size of the primer pair; (iii) the number of *in silico* PCR product(s) in the assembled genomic sequences of the sequenced *Brassica* species (currently for *B. rapa*, *B. oleracea* and *B. napus*) and (iv) the polymorphism survey and number of fragment(s) amplified in six representative *B. napus* cultivars/inbred lines.

## 4. Discussion

### 4.1. The pattern of microsatellite distribution is likely conservative in *Brassica*

In the current study, almost all of the important characteristics of microsatellite distribution in the assembled genomic sequences of the three recently sequenced *Brassica* crop species have been analysed and compared. To the best of our knowledge, this study is the first report on the genome-wide analysis and comparison of the pattern of microsatellite distribution across the different species within the same genus in plants.

First, the frequencies of microsatellites in the assembled genomic sequences of *B. rapa* (496.8 per Mb), *B. oleracea* (424.8 per Mb) and *B. napus* (420.6 per Mb) were similar, and all were higher than almost all of the previous estimations.^20,21,24,26,42^ The slightly higher frequency of microsatellites in *B. rapa* than in both *B. oleracea* and *B. napus* is likely attributable to the more concentrated distribution and lower content of TEs in the assembled genomic sequences of *B. rapa* than in *B. oleracea* and *B. napus* (Fig. 3) because the frequencies (285.5, 272.0 and 285.4 per Mb) of microsatellites in the coding DNA sequences of the three species are almost equal.^49^ Secondly, in accordance with the high correlation between these variables (Supplementary Table S1), the distributions with respect to the motif length, type and repeat number of microsatellites in the assembled genomic sequences of the three *Brassica* species were almost identical (Fig. 1; Supplementary Table S2). More specifically, both the dominant/major and absent/scarce mono- to hexanucleotide motifs in the assembled genomic sequences of the three *Brassica* species were mostly identical (Table 1; Supplementary Table S3). Interestingly, the dominant/major motifs were all A/T rich, while the absent/scarce motifs were mostly C/G rich, which corresponded well to the much higher A/T than C/G content in the analysed sequences. Thirdly, the repeat numbers of the corresponding repeats or motifs for the three *Brassica* species were mostly similar (Table 1; Supplementary Table S4). Fourthly, the total repeat length (=microsatellite number × motif length × motif repeat number) proportions of the corresponding repeats or motifs of microsatellites in the assembled genomic sequences of the three *Brassica* species were also mostly similar (Table 1; Supplementary Table S5). In addition, the genomic distributions of microsatellites in the assembled pseudochromosomes of *B. rapa* and *B. oleracea* were generally similar (Fig. 3).

In conclusion, almost all of the analysed important characteristics of microsatellite distribution in the assembled genomic sequences of the three sequenced *Brassica* crop species were highly similar or almost identical, which suggests that the pattern of microsatellite distribution is likely conservative in *Brassica*. This circumstance is understandable because *B. napus* (AACC, 2n = 38) originated from the chromosome doubling after the very recent (≈0.01 MYA) natural hybridization between *B. rapa* (AA, 2n = 20) and *B. oleracea* (CC, 2n = 18),^11^ which diverged from a common ancestor only ≈5 MYA.^50^

### 4.2. Usefulness of the newly developed genome-wide *Brassica* SSR markers

In the current study, a total of 115 869, 185 662 and 356 522 SSR markers were successfully developed from the assembled genomic sequences of *B. rapa*, *B. oleracea* and *B. napus*, respectively (Table 3), with the frequencies of 408.2, 343.8 and 356.2 per Mb or one every 2.45, 2.91 and 2.81 kb. To the best of our knowledge, this study is the first report on genome-wide SSR marker development in *Brassica*. Only a small proportion of the newly developed genome-wide SSR markers (5.2, 6.6 and 6.7% for *B. rapa*, *B. oleracea* and *B. napus*, respectively) were involved in the previously developed publicly available *Brassica* SSR markers (Supplementary Table S7), which suggests that most of the newly developed genome-wide SSR markers should represent the new SSR markers. The huge-number and high-frequency genome-wide SSR markers developed from the sequenced *Brassica* species in this study could be useful for many studies that require large-number and/or high-density molecular markers, such as high-density linkage map construction, gene/QTL fine mapping and genome-wide/regional association mapping.

The acute physical positions of the majority of the newly developed genome-wide SSR markers of the sequenced *Brassica* species have been determined (http://oilcrops.info/SSRdb) based on the mapped sequence scaffolds (Supplementary Table S6) from which they are designed. In fact, the physical positions of most of the previously developed publicly available *Brassica* SSR markers have also been determined by *in silico* mapping against the pseudochromosomes of these sequenced *Brassica* species (http://oilcrops.info/SSRdb). The high-density SSR marker-based physical maps constructed in this study could be useful for the rapid selection of genome-wide SSR markers that are well distributed over these chromosomes for various genotyping applications.

Because of the polyploidy nature of *Brassica*,^45^ the developed SSR markers usually amplify multiple fragments from the homologous DNA sequences, as revealed in the current (Supplementary Table S8) and previous^12–14,22,24,26,27,29,30,42,51^ studies in *Brassica*. This could complicate or cause errors in the genotype scoring due to the reciprocal overlapping and uncertain allelism of these fragments.^33^ However, only a small proportion of the previously developed publicly available *Brassica* SSR markers have been alleged to be single locus.^33^. Therefore, there is an urgent need to develop more single-locus SSR markers to facilitate their application in *Brassica*. Previously, the single-locus SSR markers were developed by practical PCR amplification in a panel of inbred lines,^33^ which was time consuming, labour intensive, high cost and, thus, inefficient. In the current study, through the highly efficient *in silico* PCR analysis, a large number of newly developed genome-wide SSR markers (92 517, 121 169 and 93 084 for *B. rapa*, *B. oleracea* and *B. napus*, respectively) were found to generate one *in silico* PCR product in the assembled genomic sequences of the three sequenced *Brassica* species (Table 3). In addition, thousands of previously developed publicly available *Brassica* SSR markers were also found to generate one *in silico* PCR product in the assembled genomic sequences of these *Brassica* species (http://oilcrops.info/SSRdb). More importantly, most (88.4%) of the tested SSR markers, that generated one *in silico* PCR product in the assembled genomic sequences of *B. napus*, also amplified a single clear fragment in the six representative *B. napus* cultivars/inbred lines (Supplementary Table S8). These results suggest that SSR markers that generate one *in silico* PCR product should be the putative single-locus markers and could be especially useful. Interestingly, the proportion (27.9%) of the newly developed genome-wide *Brassica* SSR markers (Table 3), which generated one *in silico* PCR product in the assembled genomic sequences of *B. napus*, was close to the corresponding proportion (33.8%) of the previously developed 9858 SSR marker from the GSSs/unique transcripts of *B. napus*, the BACs of *B. rapa* and the GSSs of *B. oleracea*,^33^ which amplified a single clear fragment in six *B. napus* inbred lines.

Also known as ‘functional’ markers,^52^ genic SSR markers are developed from genes and have a high transferability across related species.^52^ Although several studies have been conducted to develop genic SSR markers from the ESTs/unique transcripts of *B. rapa*,^29,31,32^ *B. oleracea*^31^ and *B. napus*,^30–32^ the total number (<5000) of publicly available genic SSR markers has remained limited in *Brassica* (http://oilcrops.info/SSRdb). In the current study, a large number of newly developed genome-wide SSR markers (32 648, 33 228 and 58 952 for *B. rapa*, *B. oleracea* and *B. napus*, respectively) were involved in the annotated genes (Supplementary Table S7) and thus belonged to the genic SSR markers. Of these, only a small proportion (7.2, 6.1, 6.7% for *B. rapa*, *B. oleracea* and *B. napus*, respectively) was involved in the previously developed publicly available *Brassica* SSR markers (http://oilcrops.info/SSRdb). This finding suggests that most of these newly developed *Brassica* genic SSR markers could represent the new ‘functional’ markers, which should be highly useful in evolutionary studies,^29^ comparative mapping,^32^ candidate gene association mapping^53^ and molecular breeding.

For the high transferability of SSR markers across the cultivated and wild *Brassica* species,^27,33,54,55^ the developed genome-wide SSR markers from *B. rapa* (AA, 2n = 20), *B. oleracea* (CC, 2n = 18) and *B. napus* (AACC, 2n = 38) should also be useful for *B. nigra* (BB, 2n = 16), *B. juncea* (AABB, 2n = 36), *B. carinata* (BBCC, 2n = 34) and other *Brassica* species. In addition, according to the previous marker transferability research,^15,16,22,25^ a considerable proportion of the newly developed genome-wide *Brassica* SSR markers (especially the genic SSR markers) should also be useful for the species that belong to other genera and tribes within the *Brassicaceae* family.

More importantly, we also constructed an integrative SSR marker database for *Brassica* (http://oilcrops.info/SSRdb), which not only provides useful information on the newly developed genome-wide SSR markers from the sequenced *Brassica* species (currently only for *B. rapa*, *B. oleracea* and *B. napus*) but is also integrated with the previously developed publicly available *Brassica* SSR markers and the annotated genome components (mainly as genes and TEs). To the best of our knowledge, this is the first comprehensive SSR marker database for *Brassica* until now, and it should be a significant contribution to the *Brassic*a research community.

### 4.3. Implications for SSR marker development

The numbers of clear fragment(s) amplified in the six representative *B. napus* cultivars/inbred lines for the 3974 tested SSR markers were usually equal or close to the numbers of *in silico* PCR product(s) in the assembled genomic sequences of *B. napus* (Supplementary Table S8). This finding suggests that the number of products amplified by SSR markers can be relatively accurately estimated by *in silico* PCR, which was in accordance with the previous reports in plants such as rice^56^ and *Brachypodium*.^38^ Therefore, the target microsatellite should be subjected to BLAST/*in silico* PCR analysis to estimate its copy number before SSR marker development, especially for the polyploidy species. In addition, most (88.4%) of the tested SSR markers that generate one *in silico* PCR product were also confirmed by practical PCR analysis (Supplementary Table S8). Therefore, the *in silico* identified single/low copy microsatellites should be preferential for marker development.

Replication slippage and recombination are currently two major mechanisms that are responsible for microsatellite expansion or contraction.^2,3,5,57^ Because of the small numbers of the tested SSR markers of specific motif length(s), the relationship between the SSR marker polymorphism level and the motif length was usually inconsistent in both the current (Table 4) and previous^20,24,26,30,37^ studies. However, the general trend was similar: the SSR marker polymorphism level tended to decrease as the motif length increased. This relationship is understandable because shorter motifs allow more possible replication slippage events per unit length of DNA.^58,59^ In addition, the SSR marker polymorphism level was positively correlated with both the motif repeat number and the repeat length in both the current (*r* = 0.74 and 0.86, respectively) and previous^37,47,48,60^ studies. More importantly, the tested compound SSR markers were almost all (97.6%) polymorphic. These relationships are also understandable because more motifs, larger motif repeat number and longer repeat length give more opportunity for replication slippage.^2^ Therefore, microsatellites with a shorter motif length, larger motif repeat number, longer repeat length and especially the compound repeat should be preferential for marker development.

It should be noted that a considerable proportion (Supplementary Table S7) of the newly developed genome-wide SSR markers from the sequenced *Brassica* species were involved in the so-called ‘mobile DNA sequences’ TEs^61^ and should thus be unstable. In addition, the SSR markers that are associated with TEs (especially retrotransposons) mostly generated tens to thousands of *in silico* PCR products (http://oilcrops.info/SSRdb). Therefore, caution should be observed with respect to marker development based on microsatellites that are associated with TEs (especially retrotransposons).

## Supplementary Data

Supplementary Data are available at www.dnaresearch.oxfordjournals.org.

## Funding

This work was supported by the National Science and Technology Supporting Program (2010BAD01B02), the National Rapeseed Industry Technology System (CARS-13) and the Hubei Agricultural Science and Technology Innovation Center of China.

## Supplementary Material

Supplementary Data
