# Genome-Wide Microsatellite Characterization and Marker Development in the Sequenced *Brassica* Crop Species

**DOI:** 10.1093/dnares/dsu011

**Published:** 2014-04-03

**Authors:** Jiaqin Shi, Shunmou Huang, Jiepeng Zhan, Jingyin Yu, Xinfa Wang, Wei Hua, Shengyi Liu, Guihua Liu, Hanzhong Wang

DNA RESEARCH 21, 53–68, (2014); doi:10.1093/dnares/dst040

There was an error in the first three rows of Table 1 for the column *B. oleracea* Repeat number: the 3 should be 12. The corrected part of the table is given below.

**Table 1.** Number, repeat number and total repeat length of the mono- to hexanucleotide repeats or motifs of microsatellites in the assembled genomic sequences of *B. rapa, B. oleracea* and *B. napus*

Motif	*B. rapa*			*B. oleracea*		
	Number *(%)*	Repeat number	Total length *(%)*	Number *(%)*	Repeat number	Total length *(%)*
Mono	31 258 (22.2)	12–307 (14.7)	458 968 (20.1)	55 433 (24.2)	12–65 (15.1)	838 104 (24.1)
A	29 536 (20.9)	12–50 (14.5)	428 733 (18.7)	52 021 (22.7)	12–65 (15.0)	780 171 (22.5)
C	1722 (1.2)	12–307 (17.6)	30 235 (1.3)	3412 (1.5)	12–63 (17.0)	57 933 (1.7)

