# Supplementary material for: Genome-Wide Microsatellite Characterization and Marker Development in the Sequenced Brassica Crop Species
Source: DNA Res. 2013 Oct 14;21(1):53–68. doi: 10.1093/dnares/dst040 (PMC3925394; doi:10.1093/dnares/dst040)
Supplement: Supplementary Data [file supp_21_1_53__index.html]

Genome-Wide Microsatellite Characterization and Marker Development in the Sequenced Brassica Crop Species — Genome-Wide Microsatellite Characterization and Marker Development in the Sequenced Brassica Crop Species — Supplementary Data 

# Genome-Wide Microsatellite Characterization and Marker Development in the Sequenced *Brassica* Crop Species

## Supplementary Data

Supplementary Data

**Files in this Data Supplement:**

- Supplementary Table 1 - xls file
- Supplementary Table 2 - xls file
